# Maximum extent and decay of the Laurentide Ice Sheet in Western Baffin Bay during the Last glacial episode

**DOI:** 10.1038/s41598-017-11010-9

**Published:** 2017-09-06

**Authors:** Etienne Brouard, Patrick Lajeunesse

**Affiliations:** Centre d’études nordiques & Département de géographie, Université Laval, Québec, Québec, Canada

## Abstract

Reconstructing the extent, flow and decay of the Laurentide Ice Sheet (LIS) on the continental shelves of North America during the last glaciation provides paleoglaciological analogues that are essential for understanding and predicting how modern marine-based ice-sheets will respond to future climate change and sea level fluctuations. The geometry of the LIS during Marine isotope stage 2 (MIS-2; 29–14 ka BP) is one key element for ice-sheet modelling. The maximum extent of the LIS during this stage is well constrained for most sectors of the ice sheet, but major uncertainties remain, especially along the continental shelves of Arctic Canada. Despite a series of recent papers, the extent of the LIS in Western Baffin Bay, an area draining large volumes of glacial ice through multiple ice streams and likely characterized by ice shelves, remains highly speculative. Here we present unequivocal marine geophysical evidence that during the MIS-2 the LIS extended to the edge of the continental shelf, seaward of the previously proposed limits and subsequently retreated episodically westward during deglaciation. These data support interpretations of deep glacial ice grounding in Western Baffin Bay.

## Introduction

**Figure 1:**
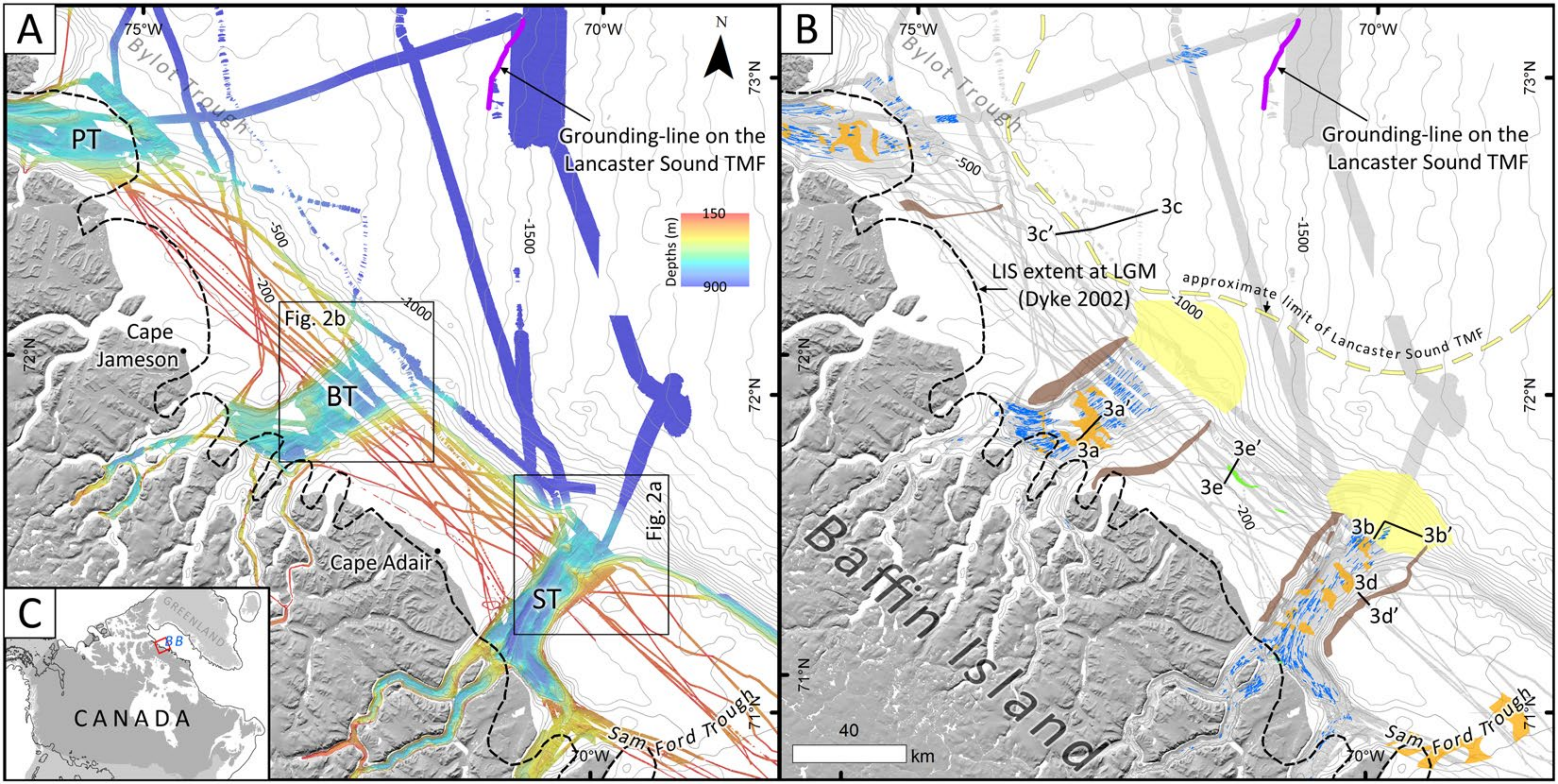
(**A**) The map shows the bathymetric data collected by the ArcticNet program on the northeastern Baffin Island shelf. The black dashed-line shows the widely accepted maximum extent of the LIS margin during MIS-2. ST: Scott Trough; BT: Buchan Trough; PT: Pond Trough. (**B**) Geomorphological map of glacial landforms and deposits in the study area. Blue lines represent the MSGLs; brown areas represent ISLMs; orange areas represent GZWs; light yellow areas represent the extent of TMFs; and the green areas represent transverse ice-contact ridges. Profiles shown on Fig. 3 are located by black lines. Light-gray lines: 100 m contours from the International Bathymetric Chart of the Arctic Ocean database^54^. (**C**) Location of the study area and maximum extent of the LIS at LGM according to Dyke (2004). BB: Baffin Bay. Maps were produced in ESRI ArcGIS 10.4 (http://www.esri.com) and edited in Adobe Photoshop CS5 (www.adobe.com/photoshop).

The maximum extent of the LIS in Western Baffin Bay during MIS-2 has been a subject of debate for decades and brought different glaciation scenarios oscillating between extensive single dome LIS reaching the edge of the continental shelf ^1^ and a minimal ice hypothesis where the LIS only reached the fjords head^2, 3^. The Flint (extensive) scenario^1^ was supported by modelling^4^ but, however, lacked field data while the minimalist scenario has been challenged by recent dating of moraines^5, 6^ and by the identification of terrain preserved under cold-based ice on coastal forelands^7, 8^. Cosmogenic nuclides exposure dating on terrestrial moraines as well as lacustrine and marine sediment cores data were used to resolve the enigma of the maximum extent of the LIS by proposing a scenario where most of LIS outlet glaciers reached the continental shelf at fjord mouths, with ice margins terminating inland in-between fjords (i.e., the Goldilocks Paradigm)^9^. This interpretation was largely used in most paleogeographical reconstructions and in ice-sheet modelling studies^10–15^. More recent studies have, however, challenged this model, suggesting a more extensive LIS margin on the northeastern Baffin Island shelf during the Last Glacial Maximum (LGM) based on the observation of (1) an ice-contact seismostratigraphic unit extending on most of the continental shelf (the Baffin Shelf Drift^16^) and (2) a till wedge at 1300 m water depths at the southeast end of Lancaster Sound deposited at the ice grounding-zone^17^ (Fig. 1). Therefore, new constraints are needed to enable a more rigorous representation of the LIS extent in the region in order to improve the detail of paleoglaciological models. Swath bathymetry imagery collected over a period of 13 years during scientific expeditions onboard the CCGS Amundsen combined with archived seismic reflection data from the Geological Survey of Canada are used here to map submarine glacial landforms in order to reconstruct the extent, flow and retreat of the LIS margin on the northeast Baffin Island shelf during MIS-2.

## Glacial imprints

### Cross-shelf troughs and ice-flow features

**Figure 2:**
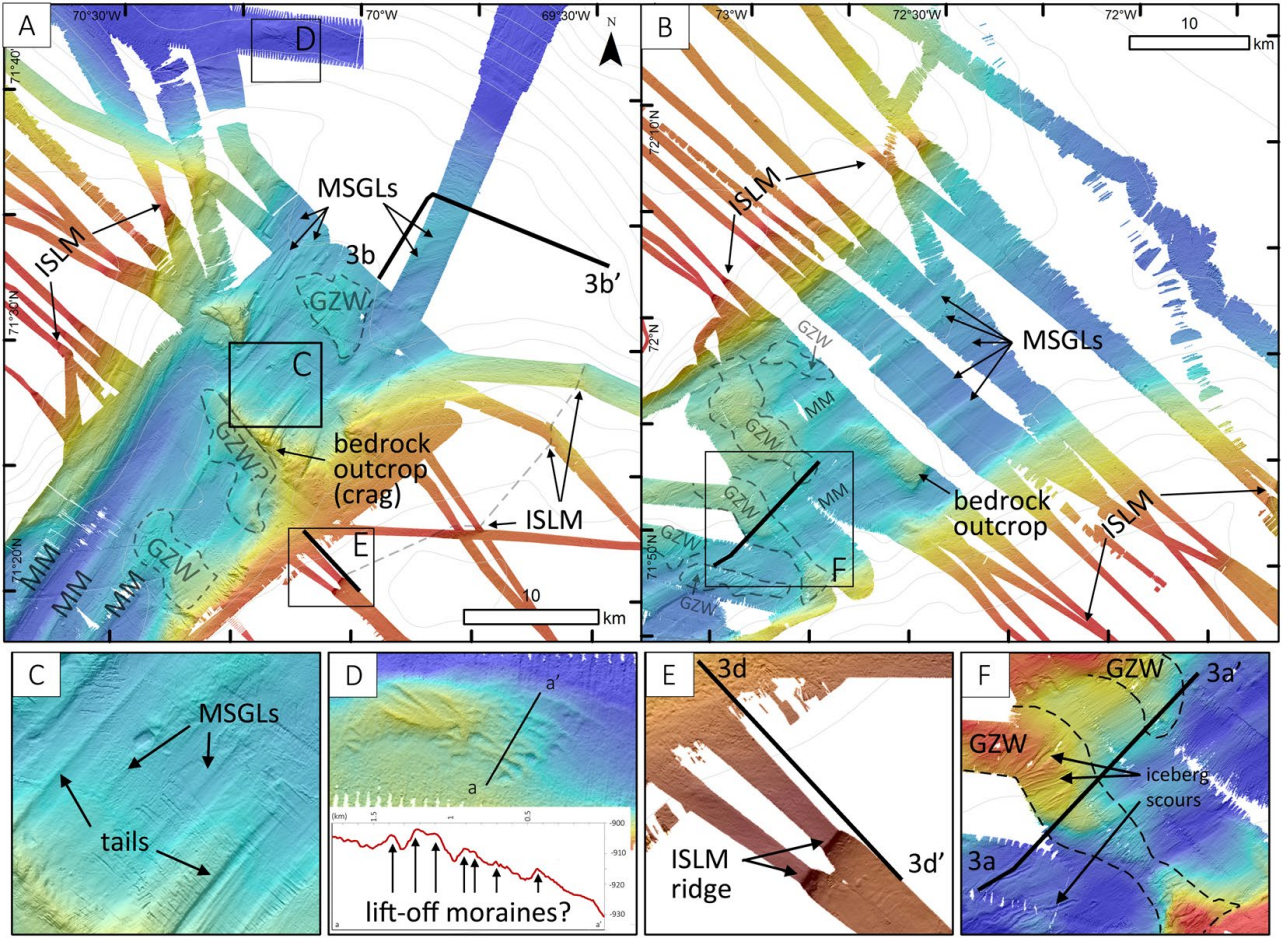
Bathymetry and examples of glacial landforms in Scott (**A**) and Buchan (**B**) troughs. (**C**) Examples of crag-and-tails and MSGLs in outer Scott Trough. (**D**) Possible lift-off moraines at the shelf break on Scott TMF. (**E**) ISLM south ridge of Scott Trough. (**E**) GZW overprinted by iceberg scours in Buchan Trough. MM: medial moraine; GZW: Grounding-zone wedge; MGSL: Mega-scale glacial lineation; ISLM: Ice stream lateral moraine. Swath bathymetry is at a 10 mpp resolution. Black lines show the location of seismic profiles shown on Fig. 3. Maps were produced in ESRI ArcGIS 10.4 (http://www.esri.com) and edited in Adobe Photoshop CS5 (www.adobe.com/photoshop).

Four cross-shelf troughs that were glacially eroded and moulded during Quaternary glaciations^16, 18^ are observed offshore major fjord systems of northeastern Baffin Island: Pond, Buchan, Scott and Sam Ford troughs (Fig. 1A). These major submarine valley systems alone, however, do not provide unequivocal evidence for ice-flow during last glacial cycle, as they mostly reflect a long-term record of repeated stages of glacial erosion that probably occurred since the Pliocene. Nonetheless, assemblages of smaller-scale ice-flow landforms are observed within Buchan, Scott and Pond troughs on the swath bathymetry imagery. Ice-flow morphologies include mega-scale glacial lineations (MSGLs), ice stream lateral moraines (ISLMs), medial moraines, crag-and-tails, drumlins, meltwater channels and grooves^19, 20^ (Figs 1 B and 2). MSGLs are indicators of fast ice-flow and are a common and unequivocal signature of ice stream activity^21, 22^. These elongated landforms (>450 m lengths; <250 m widths; elongation ratios >10:1) are aligned in the trough axis and have low amplitudes (<20 m). Their very good state of preservation and the weak attenuation of their morphology due to a thin cover of postglacial sediments suggest that they relate to the last stage of ice occupation. Crosscut by grounding-zone wedges, MSGLs are interpreted to reflect time-transgressive ice-flows occurring during the landward retreat of an ice stream. In Pond and Scott troughs, MSGLs extend onto the shelf break (Figs 1 B and 2), while in Buchan Trough, the lack of extensive swath bathymetry coverage makes it difficult to determine the seaward extent of the MSGLs (Fig. 2B). A small set of MSGLs lies at ~1100 m present-day water depths some 75 km east of Pond Trough (Fig. 1B). These landforms are oriented (ESE) towards the grounding-zone deposit interpreted by Li *et al*. (2011) as the limit of the LIS margin during MIS-2.

### Grounding-zones wedges at the ice margin

**Figure 3:**
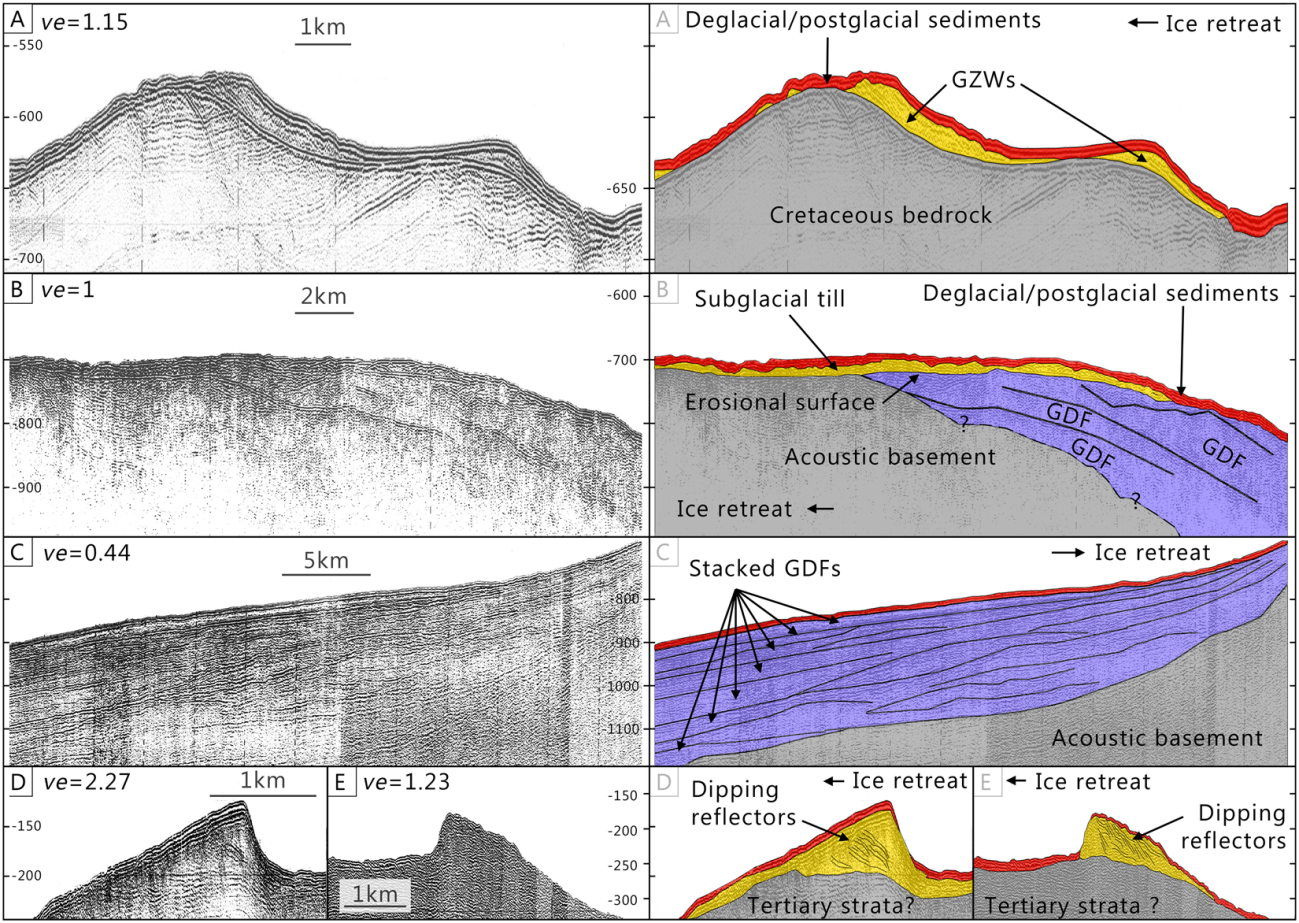
Examples of sediments architecture on seismic reflection (Airgun) profiles. Left-side panels show the contrast-brightness enhanced profiles while right-side panels show the interpretation of the profiles. (**A**) Grounding-zone wedges (GZWs) in Buchan Trough. (**B**) Stacked glacial debris flows (GDFs) topped by a till unit with an erosional surface. (**C**) Stacked GDFs on the Lancaster Trough-mouth fan. (**D**) Lateral marginal moraine with dipping reflectors in outer Scott Trough. (**E**) Ice-contact fan (moraine) with seaward dipping reflectors, between Scott and Buchan Trough. See Figs 1 and 2 for profile locations. *ve*: vertical exaggeration. Profiles were acquired using airguns by the Geological Survey of Canada (Profile A: Line 78029_AG_265_0816; Profile B: line 78029_AG_267_0155; Profile C: 78029_AG_271_2300; Profile D: 76028_AG_248_1534; Profile E: 80028_AG_EPC1_254_0825). Seismic reflection data were analyzed and extracted using the LizardTech GeoViewer software (https://www.lizardtech.com/geoviewer-pro/overview). Maps and seismic reflection data were transferred to the Adobe Photoshop CS5 software (www.adobe.com/photoshop) for figure production and editing.

Grounding-zone wedges (GZWs) are formed by the accumulation of subglacial sediments at the grounding zone of an ice stream during temporary standstills of an ice margin e﻿.g. 23–25. GZWs have been associated with the presence of ice shelves^24^ which restricts vertical accommodation space for sediments in favor of sediment progradation, leading to the formation of low-amplitude and horizontally extensive landforms (i.e., GZWs)^23^. GZWs are observed in the four studied troughs from swath bathymetry imagery and/or seismic data (Figs 1 B, 2 and 3A). On swath bathymetry imagery, GZWs form asymmetric tabular topographic highs that are perpendicularly aligned to trough orientations and characterized by stoss sides with lower gradients (Fig. 3A). On seismic reflection profiles, most of the GZWs consist of acoustically semi-transparent to chaotic wedge-shaped deposits overlain by thin unit of high-amplitude parallel reflections (Fig. 3A). The transparent to chaotic acoustic unit is interpreted as diamictic debris, mainly from a sub-ice stream deformable till layer^26–29^ delivered to the grounding zone during a period of ice margin stabilization^24^. GZWs are conformably covered by a thin sediment drape (6–8 m) that is coherent with postglacial sedimentation rates observed in Scott Trough^16^. Also, all the GZWs observed in Scott, Buchan and Pond troughs overprint sets of MSGLs formed during a previous phase of ice-flow (Fig. 2). They are therefore interpreted to reflect episodic phases of ice margin stabilization during deglaciation rather than a maximum position^30^.

### Trough-mouth fans and glacigenic debris flows

At the shelf edge of Scott Trough, MSGLs terminate at the head of a 17 by 42 km-wide fan-shaped bathymetric bulge interpreted as a trough-mouth fan (TMF)^17, 31–33^. TMFs generally consist of stacked acoustically transparent lenses of glacigenic-debris flows (GDFs) separated by acoustically stratified units representing suspension-settling sediments, turbidites and/or contourites^34–37^. GDFs are mostly the product of instabilities at the grounding zone during the occupation of the trough by an ice stream, while the stratified units relate to deglacial to interglacial conditions^17, 38–43^. Stacked GDFs interbedded with stratified units observed on seismic reflection profiles in Scott Trough could represent such a succession of glacial debris flows and interglacial sedimentation, but of unknown age (Fig. 3B). At the distal end of the trough, an erosional surface truncates the upper stacked GDFs. This surface is overlain by an acoustically semi-transparent unit interpreted as till, which is itself overlain by a thin unit (~6–8 m) of high amplitude parallel reflections interpreted as glaciomarine and hemipelagic sediments^16^. The erosional surface and the above sediment sequence indicate a glacial advance followed by deglacial to postglacial sedimentation. This sequence is also observed in the outer sector of Buchan Trough in a similar 18 by 44 km TMF. No similar bathymetric bulge is present at the mouth of Pond Trough, as the latter ends abruptly at its junction with Bylot Trough. Seismic reflection data also show stacked GDFs at the southern end of the Bylot Trough on the Lancaster Fan, just east of Cape Jameson Bank (Fig. 3C). A hummocky terrain and undulating ridges are identified at the shelf edge on Scott TMF (Fig. 2A). These landforms are similar to ‘lift-off moraines’ and could reflect temporary stabilisation of the grounding-zone of a tidally influenced ice margin^44^. Consequent with the extent of the till unit on the TMF, these ridges probably reflect the maximal extent of the LIS margin in Scott Trough.

### Ice stream lateral moraines

Ice stream lateral moraines (ISLMs) indicate fast-flowing ice surrounded by either slower ice-flow or ice-free terrain^45, 46^. Multiple ISLMs are observed on both sides of Buchan and Scott troughs (Fig. 1B and 2), forming >40 km linear ridges with a relief reaching 120 m. On seismic reflection profiles, the outer-trough moraines show prograding internal reflections dipping away from the trough (Fig. 3D). This dipping architecture within the outer-trough ISLMs reflects horizontal sediment progradation, in a similar way to GZWs formation^47^. Sediment progradation therefore indicates the absence of horizontal constraints (i.e., grounded ice) on the outer continental shelf. Similarly to GZWs it could also indicate the presence of a constraint for vertical accumulation (i.e., ice shelf)^47^.

### Ice-contact sediments on the continental shelf

Swath bathymetry imagery reveals ridges parallel to the shelf break on the banks seaward of Cape Adair (Fig. 1B). These ridges consist of acoustically semi-transparent sediment bodies overlain by a thin (<10 m) unit of high amplitude parallel reflections (Fig. 3E). Based on their architecture and amplitude, these ridges are interpreted as ice-contact fans or moraine ridges typical of tidewater glaciers on high-latitude continental shelves^48^. The chaotic acoustically semi-transparent unit extends laterally from the ridges and parallel to the shelf break; it is interpreted as ice-contact sediments correlated to the Baffin Shelf Drift^16^.

## Extent of the Laurentide Ice Sheet

### Position and timing

**Figure 4:**
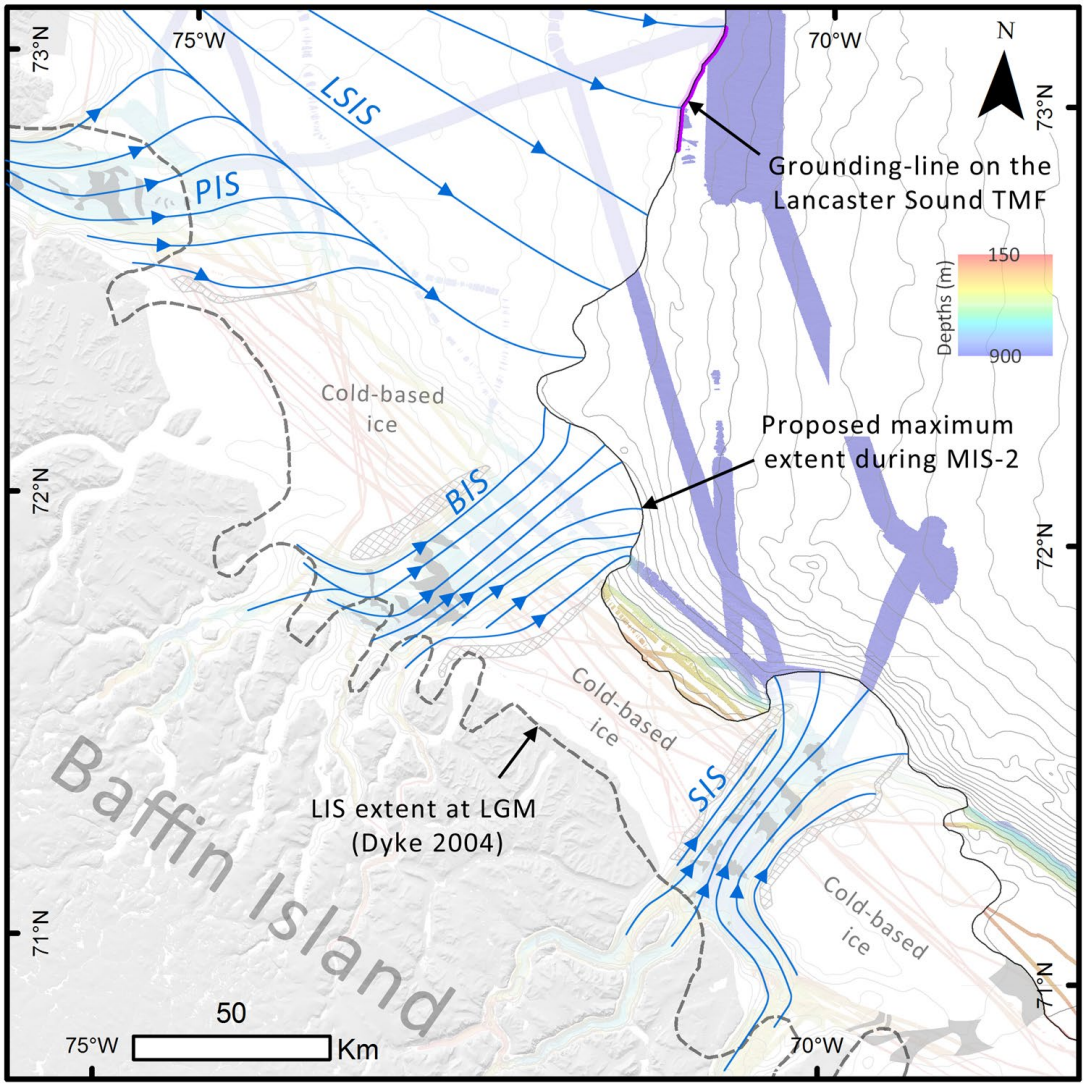
Maximum extent of the Laurentide Ice Sheet on the northeastern Baffin Island continental shelf during MIS-2 and inferred ice streams. Blue lines represent ice-flow lines within ice streaming areas. LSIS: Lancaster Sound Ice Stream; PIS: Pond Ice Stream; BIS: Buchan Ice Stream; SIS: Scott Ice Stream. Gray areas within troughs represent the GZWs; the crosshatch areas represent the ISLMs. Light-gray lines: 100 m contours from the International Bathymetric Chart of the Arctic Ocean database^54^. Maps were produced in ESRI ArcGIS 10.4 (http://www.esri.com) and edited in Adobe Photoshop CS5 (www.adobe.com/photoshop).

Ice-contact landforms and deposits mapped on the northeastern Baffin Island continental shelf allow reconstructing the configuration of the maximum extent of the LIS during MIS-2. In Scott and Buchan troughs, the presence of MSGLs extending to the shelf break, together with a till unit extending on the TMFs, indicate that the LIS reached the shelf break during the last glacial episode (Fig. 4). The GZWs observed in the troughs are located landward from the maximal extent of the LIS at the shelf edge. Therefore, they are interpreted to have been constructed during phases of standstill of the westward episodically retreating LIS margin. MSGLs mapped in Pond Trough reach Bylot Trough, but the absence of a depocenter (TMF) indicates that the LIS extended farther in this area. The LIS grounding-zone at the end of Lancaster Sound^9^ is located ~100 km eastward from the MSGLs terminus. Ice flowing through Pond Trough likely converged into the Lancaster Ice Stream in Bylot Trough and from there flowed eastward to reach the LGM grounding-zone on the Lancaster TMF^17^. Subglacial sediments delivered to Bylot Trough by the Pond Trough ice stream would have been taken in charge by Lancaster ice that fed the Lancaster Fan. In Sam Ford Trough, GZWs constructed from the same till/ice-contact seismic unit in both Buchan and Scott troughs extends at least to the middle of Sam Ford Trough to form a distinct transverse-to-flow ridge (Fig. 1B). This northeast GZW is the farthest confirmed position of the LIS in Sam Ford cross-shelf trough. It cannot be ruled out, however, that the LIS margin reached the shelf break at the mouth of Sam Ford Trough as geophysical data are needed farther offshore.

On the inter-trough areas between Sam Ford and Buchan troughs, the Baffin Shelf Drift and the position of the moraines extend near the shelf break. These ice-contact sediment bodies are geometrically linked to the seaward extent of the ISLMs. They provide evidence for a continuous maximum margin between Sam Ford Trough and Buchan Trough. Offshore Cape Jameson, the inter-trough area separating Pond and Buchan troughs was probably covered by ice, but additional data is required to enable the identification of ice-contact sediments. GDFs observed east of the Cape Jameson Bank reflect ice flowing from the Lancaster Ice Stream trough Bylot Trough.

Ice-contact deposits observed at the shelf edge are interpreted as MIS-2 because (1) they occur down-ice (eastward) from series of GZWs deposited during an episodic westward retreat of the LIS margin; and (2) the grounding-zone wedges are overlain by only one sedimentary sequence of deglacial-postglacial deposition corresponding to the last glacial retreat. The thickness of the upper sedimentary sequence along with the sedimentation rates observed in Scott Trough support a deglacial origin for the GZWs. A deglacial age for the GZWs therefore implies a seaward position of the LIS margin during MIS-2. The LIS margin could have reached its maximum extent prior to the LGM^49^ and remained at this position until well after the LGM. Extrapolation of radiocarbon dates on postglacial sediments in Scott Trough yield a minimum age of 15 ka BP for the beginning of postglacial sedimentation and, therefore, for the end of glaciation on the shelf^16^. We can only presume that the maximum extent of the LIS represents the LGM conditions during MIS-2, but further investigations including absolute chronology dating should focus on constraining the exact timing of the maximum ice extent. An absolute age for the LGM on the northeast Baffin Island continental shelf is not well constrained because: (1) the ice-contact sediments and the TMFs reported here could be of diachronic origin; (2)^14^C dates from these deposits are lacking; and (3) the LGM is a diachronic position corresponding to a time-period rather than a precise event in time. This time-period along eastern Baffin Island is yet to be resolved^50^.

### Ice dynamics

The revised ice-margin and the distribution of highly elongated bedforms (MSGLs) indicate that ice streams were operating along Buchan, Scott and Pond troughs during MIS-2. The presence of ISLMs suggests that the LIS was experiencing spatial variations in ice-flow velocities, suggesting that inter-trough areas, were submitted to slow ice-flow or cold-based ice^45, 46, 51–53^. The presence of cold-based ice in the inter-ice stream areas is in accordance with the preservation of older deposits than MIS-2 on coastal forelands in between the fjords^7^. The basal thermal regime of the LIS in the region seems therefore complex and spatially variable with low-gradient outlet fast-flowing ice being constrained to the fjords axis as proposed by Miller *et al*. (2002). In Sam Ford Trough, the absence of ice-flow morphologies and the presence of perpendicular-to-trough ridges suggest that ice-flow was slow in the trough. This slow ice-flow was probably due to the transport of ice discharge from Sam Ford Fjord into the Scott Inlet Ice Stream. The ice-flow route from Sam Ford Fjord to Scott Inlet can be inferred from the northward orientation of MSGLs and drumlins at the junction of Sam Ford Fjord and Hecla & Griper Trough. The location of the GZWs suggests that the retreat of the LIS margin from the shelf edge to the fjords head has been marked by several phases of stabilization.

## Conclusions

The swath bathymetry and geophysical data presented in this paper provide firm evidence that the LIS extended across the northeast Baffin Island continental shelf during MIS-2, reaching the continental shelf break between Scott Trough and Pond Trough. These data also confirm the presence of grounded glacial ice on the Lancaster Fan east of Pond Trough during MIS-2. The proposed maximum extent of the LIS during MIS-2 is located at least 36 km seaward of the previously proposed limits^9, 50^ and is in accordance with inferred LGM extents recently raised^16–18^.

Fast-flowing ice streams occupied Pond, Buchan and Scott troughs while slow ice-flow or cold-based ice occupied the shelf and Sam Ford Trough. The presence of GZWs in the four major cross-shelf troughs indicates an episodic retreat style punctuated by ice-margin stabilization rather than a rapid/catastrophic retreat^16^. Finally, these results indicate that the LIS deglaciation was driven by a complex spatially varying thermal regime with low-gradient ice-streams in the troughs and cold-based/slow-flowing ice on the inter-trough areas. An LIS deglaciation from an outer seaward position implies differences in the LIS geometry, dynamics, flow-mechanisms and feedbacks to post-LGM relative sea-level rise in Western Baffin Bay. This revised ice margin and its retreat dynamics should therefore be taken into account to increase the accuracy of LIS reconstructions or modelling, which have implications on how we can understand the long-term evolution of a marine-based ice-sheet during deglaciation.

## Methods

Multibeam data were collected using Kongsberg Simrad EM-300 (12 kHz) and EM-302 (30 kHz) echosounders onboard the CCGS Amundsen by the Ocean Mapping Group (University of New Brunswick) and the Laboratoire de Géosciences Marines (Université Laval) for the ArcticNet program. The multibeam data were processed using the Caris HIPS&SIPS (www.caris.com/products/hips-sips/) and MB-System (https://www.ldeo.columbia.edu/res/pi/MB-System/) softwares. Data visualization and mapping was realized using ESRI ArcGIS 10.4 software (http://www.esri.com). Seismic reflection data were analyzed and extracted using the LizardTech GeoViewer software (https://www.lizardtech.com/geoviewer-pro/overview). Maps and seismic reflection data were transferred to the Adobe Photoshop CS5 software (www.adobe.com/photoshop) for figure production and editing. Seismic reflection data were enhanced using the Brightness/Contrast tool in Adobe Photoshop CS5 for a clearer visualization.

### Data availability

The multibeam bathymetry dataset can be visualized on the Université Laval Géoindex + website (http://geoindex-plus.bibl.ulaval.ca). The seismic reflection data along with the acquisition specifics are available on the Geological Survey of Canada website (http://ftp.maps.canada.ca/pub/nrcan_rncan/raster/marine_geoscience/Seismic_Reflection_Scanned/).

## References

[1] Flint RF (1943). Growth of North American ice sheet during the Wisconsin age. Bull. Geol. Soc. Am..

[2] Löken OH (1966). Baffin Island Refugia Older than 54,000 Years. Science (80-.)..

[3] Dyke AS, Prest VK (1987). Late Wisconsinan and Holocene History of the Laurentide Ice Sheet. Géographie Phys. Quat..

[4] Denton, G. & Hughes, T. The Last great ice sheets. John Wiley & Sons 484 (1981).

[5] Briner JP, Overeem I, Miller GH, Finkel RC (2007). The deglation of Clyde Inlet, northeastern Baffin Island, Arctic Canada. J. Quat. Sci..

[6] Young NE, Briner JP, Rood DH, Finkel RC (2012). Glacier Extent During the Younger Dryas and 8.2-ka Event on Baffin Island, Arctic Canada. Science (80-.)..

[7] Davis PT, Briner JP, Coulthard RD, Finkel RW, Miller GH (2006). Preservation of Arctic landscapes overridden by cold-based ice sheets. Quat. Res..

[8] Briner JP, Miller GH, Davis PT, Finkel RC (2006). Cosmogenic radionuclides from fiord landscapes support differential erosion by overriding ice sheets. Bull. Geol. Soc. Am..

[9] Miller GH (2002). The goldilocks dilemma: Big ice, little ice, or ‘just-right’ ice in the eastern canadian arctic. Quat. Sci. Rev..

[10] Dyke AS (2004). An outline of North American deglaciation with emphasis on central and northern Canada. Dev. Quat. Sci..

[11] Peltier WR (2004). Global Glacial Isostasy and the Surface of the Ice-Age Earth: The ICE-5G (VM2) Model and GRACE. Annu. Rev. Earth Planet. Sci..

[12] Peltier WR, Argus DF, Drummond R (2015). Space geodesy constrains ice age terminal deglaciation: The global ICE-6G-C (VM5a) model. J. Geophys. Res. Solid Earth.

[13] Stokes, C. R. & Tarasov, L. Ice streaming in the Laurentide Ice Sheet: A first comparison between data-calibrated numerical model output and geological evidence. *Geophys*. *Res*. *Lett*. **37** (2010).

[14] Tarasov L, Dyke AS, Neal RM, Peltier WR (2012). A data-calibrated distribution of deglacial chronologies for the North American ice complex from glaciological modeling. Earth Planet. Sci. Lett..

[15] Livingstone SJ, Clark CD, Tarasov L (2013). Modelling North American palaeo-subglacial lakes and their meltwater drainage pathways. Earth Planet. Sci. Lett..

[16] Praeg, D., Maclean, B. & Sonnichsen, G. *Quaternary Geology of the Northeast Baffin Island Continental Shelf*, *Cape Aston to Buchan Gulf (70*^*o*^*to 72*^*o*^*N)*. *Geological Survey of Canada Open File Report* (2007).

[17] Li G, Piper DJW, Calvin Campbell D (2011). The Quaternary Lancaster Sound trough-mouth fan, NW Baffin Bay. J. Quat. Sci..

[18] Margold, M., Stokes, C. R. & Clark, C. D. Ice streams in the Laurentide Ice Sheet: Identification, characteristics and comparison to modern ice sheets. Earth-Science Reviews **143**, 117–146 (2015).

[19] Bennett R, Campbell DC, Furze MFA, Haggart JW (2015). The shallow stratigraphy and geohazards of the Northeast Baffin Shelf and Lancaster Sound. Bull. Can. Pet. Geol..

[20] Dowdeswell EK, Todd BJ, Dowdeswell JA (2016). Submarine medial moraines and convergent ice flow, Scott Inlet, Baffin Island, Arctic Canada. Geol. Soc. London, Mem..

[21] Stokes CR, Clark CD (2002). Are long subglacial bedforms indicative of fast ice flow?. Boreas.

[22] Clark CD, Tulaczyk SM, Stokes CR, Canals M (2003). A groove-ploughing theory for the production of mega-scale glacial lineations, and implications for ice-stream mechanics. J. Glaciol..

[23] Dowdeswell JA, Fugelli EMG (2012). The seismic architecture and geometry of grounding-zone wedges formed at the marine margins of past ice sheets. Bull. Geol. Soc. Am..

[24] Batchelor CL, Dowdeswell JA (2015). Ice-sheet grounding-zone wedges (GZWs) on high-latitude continental margins. Marine Geology.

[25] Lajeunesse P (2016). Late Wisconsinan grounding-zone wedges, northwestern Gulf of St Lawrence, eastern Canada. Geol. Soc. London, Mem..

[26] Alley RB, Blankenship DD, Bentley CR, Rooney ST (1986). Deformation of till beneath ice stream B, West Antarctica. Nature.

[27] Blankenship DD, Bentley CR, Rooney ST, Alley RB (1987). Till beneath ice stream B: 1. Properties derived from seismic travel times. J. Geophys. Res. Solid Earth.

[28] Dowdeswell JA, Ó Cofaigh D, Pudsey CJ (2004). Thickness and extent of the subglacial till layer beneath an Antarctic paleo-ice stream. Geology.

[29] Christianson K (2014). Dilatant till facilitates ice-stream flow in northeast Greenland. Earth Planet. Sci. Lett..

[30] Dowdeswell, J. A., Ottesen, D., Evans, J., Ó Cofaigh, C. & Anderson, J. B. Submarine glacial landforms and rates of ice-stream collapse. *Geology* **36**, 819–822 (2008).

[31] Vorren TO, Hald M, Lebesbye E (1988). Late Cenozoic environments in the Barents Sea. Paleoceanography.

[32] Ó Cofaigh C, Taylor J, Dowdeswell JA, Pudsey CJ (2003). Palaeo-ice streams, trough mouth fans and high-latitude continental slope sedimentation. Boreas.

[33] Roger J, Saint-Ange F (2013). Late Quaternary glacial history and meltwater discharges along the Northeastern Newfoundland Shelf. Can. J. Earth Sci..

[34] Laberg JS, Vorren TO (1995). Late Weichselian submarine debris flow deposits on the Bear Island Trough Mouth Fan. Mar. Geol..

[35] Ó Cofaigh, C. *et al*. Sediment reworking on high-latitude continental margins and its implications for palaeoceanographic studies; insights from the Norwegian-Greenland Sea. *Glacier-Influenced Sediment. High-Latitude Cont. Margins* **203**, 325–348 (2002).

[36] Taylor, J., Dowdeswell, J. A., Kenyon, N. H. & Ó Cofaigh, C. S. in *Glacier-Influenced Sedimentation on High-Latitude Continental Margins* 55–71, doi:10.1144/GSL.SP.2002.203.01.04 (2002).

[37] Passchier S (2003). Pliocene-Pleistocene glaciomarine sedimentation in eastern Prydz Bay and development of the Prydz trough-mouth fan, ODP Sites 1166 and 1167, East Antarctica. Mar. Geol..

[38] Dowdeswell JA (1996). Large-scale sedimentation on the glacier-influenced polar North Atlantic Margins: Long-range side-scan sonar evidence. Geophys. Res. Lett..

[39] Vorren TO, Laberg JS (1997). Trough mouth fans - Palaeoclimate and ice-sheet monitors. Quat. Sci. Rev..

[40] King EL, Haflidason H, Sejrup HP, Løvlie R (1998). Glacigenic debris flows on the North Sea Trough Mouth Fan during ice stream maxima. Mar. Geol..

[41] Dowdeswell JA, Ó Cofaigh C, Pudsey CJ (2004). Continental slope morphology and sedimentary processes at the mouth of an Antarctic palaeo-ice stream. Mar. Geol..

[42] Wilken M, Mienert J (2006). Submarine glacigenic debris flows, deep-sea channels and past ice-stream behaviour of the East Greenland continental margin. Quat. Sci. Rev..

[43] Nygård A (2007). Extreme sediment and ice discharge from marine-based ice streams: New evidence from the North Sea. Geology.

[44] Elvenes S, Dowdeswell JA (2016). Possible ‘lift-off moraines’ at grounded ice-sheet margins, North Norwegian shelf edge. Geol. Soc. London, Mem..

[45] Dyke AS, Morris TF (1988). DRUMLIN FIELDS, DISPERSAL TRAINS, and ICE STREAMS IN ARCTIC CANADA. Can. Geogr./Le Géographe Can..

[46] Stokes CR, Clark CD (2002). Ice stream shear margin moraines. Earth Surf. Process. Landforms.

[47] Batchelor CL, Dowdeswell JA (2016). Lateral shear-moraines and lateral marginal-moraines of palaeo-ice streams. Quaternary Science Reviews.

[48] Lønne I (2001). Dynamics of marine glacier termini read from moraine architecture. Geology.

[49] Mix AC, Bard E, Schneider R (2001). Environmental processes of the ice age: Land, oceans, glaciers (EPILOG). Quaternary Science Reviews.

[50] Dyke AS (2002). The Laurentide and Innuitian ice sheets during the Last Glacial Maximum. Quat. Sci. Rev..

[51] Dyke, A. S., Morris, T. F., Green, D. & England, J. *Quaternary geology of Prince of Wales Island*, *Arctic Canada*. *Geological Survey of Canada*, *Memoir* **433** (1992).

[52] Clark CD, Stokes CR (2001). Extent and basal characteristics of the M’Clintock Channel Ice Stream. Quat. Int..

[53] Ottesen, D., Rise, L., Knies, J., Olsen, L. & Henriksen, S. The Vestfjorden-Trænadjupet palaeo-ice stream drainage system, mid-Norwegian continental shelf. *Mar. Geol.* **218**, 175–189 (2005).

[54] Jakobsson, M. *et al*. The International Bathymetric Chart of the Arctic Ocean (IBCAO) Version 3.0. *Geophys*. *Res*. *Lett*. **39** (2012).

